# The Outcomes of IgA Nephropathy With Nephrotic Syndrome: A Clinicopathological Study From Northern India

**DOI:** 10.7759/cureus.76307

**Published:** 2024-12-24

**Authors:** Abdullah, Durgesh Pushkar, Anupma Kaul, Manas R Behera, Mudit Khurana, Narayan Prasad, Dharmendra S Bhadauria, Manas R Patel, Monika Yachha, Ravi S Kushwaha

**Affiliations:** 1 Nephrology, Institute of Medical Sciences, Banaras Hindu University, Varanasi, IND; 2 Nephrology, King George's Medical University, Lucknow, IND; 3 Nephrology, Sanjay Gandhi Postgraduate Institute of Medical Sciences, Lucknow, IND; 4 Nephrology, Sarojini Naidu Medical College, Agra, IND; 5 Nephrology, Medanta Super Speciality Hospital, Lucknow, IND

**Keywords:** egfr creatinine, iga nephropathy (igan), immunosuppression therapy, mest-c score, nephrotic syndrome

## Abstract

Introduction

Nephrotic syndrome, an unusual clinical presentation of IgA nephropathy (IgAN), occurs only in a few cases. The data regarding its clinical characteristics and treatment outcomes are lacking.

Material and methods

In this retrospective analysis, we reviewed kidney biopsies conducted between January 2007 and December 2018. All patients with biopsy-proven IgAN and clinical presentation of nephrotic syndrome were included.

Results

Sixty-seven (11.9%) out of 560 patients with primary IgAN had nephrotic syndrome as the first clinical presentation. On MEST-C scoring, the baseline estimated glomerular filtration rate (eGFR) was significantly lower in the presence of segmental sclerosis (S1) (p=0.04) and >25% tubular atrophy/interstitial fibrosis (T1/2) (p=0.004). With immunosuppression, complete remission (CR), partial remission (PR), and no response (NR) occurred in 28 (41.8%), 32 (47.8%), and 7 (10.4%) patients, respectively. During the median follow-up of 190 weeks, the median absolute change in eGFR was significant between CR and NR groups (p=0.002), and PR and NR groups (p=0.02); however, it was not significant between CR and PR groups (p=0.14). In the CR, PR, and NR groups, 9, 15, and 7 patients had eGFR losses of ≥15 ml/min (p=0.004). No patient in the CR group, one in the PR group, and three in the NR group progressed to end-stage kidney disease (ESKD) (p=0.003). Further, none of the MEST-C scores correlated with the clinical outcome parameters.

Conclusion

Immunosuppression in treating IgAN accompanied by nephrotic syndrome helps in proteinuria remission. Achieving remission, whether complete or partial, delays disease progression and improves renal survival. Moreover, the baseline eGFR was significantly lower when S1 and T1/2 lesions were present in kidney biopsies.

## Introduction

Immunoglobulin A (IgA) nephropathy, or IgAN, is the most prevalent primary glomerular disease worldwide [1,2]. About 30-40% of patients develop end-stage kidney disease (ESKD) within 20 years of IgAN diagnosis, underscoring that IgAN is not benign [1,3,4]. The clinical presentation of IgAN ranges from microscopic to macroscopic hematuria, along with varying degrees of proteinuria, including nephrotic syndrome (NS), chronic kidney disease (CKD), and acute kidney injury (AKI) [5]. NS in patients with IgAN is an unusual presentation occurring in approximately 5-10% of cases [6-9]. Heavy proteinuria in IgAN patients usually indicates severe glomerular damage in advanced kidney failure [4]. However, a subset of IgAN patients with NS presentation has histologic findings of mild mesangial proliferation, with trace or 1+ IgA deposition on immunofluorescence and diffuse foot process effacement on electron microscopy [10-12]. Many of such patients are rapidly responsive to steroids [13-14]. It is unclear whether this is a variant of minimal change disease (MCD) with IgG deposits or a variant of true IgAN. Some reports have described the presence of focal segmental sclerosis in these patients with less favorable responses to steroids [15].

Thus, NS has yet to be well characterized in IgAN, and few studies have focused on IgAN patients with nephrotic-range proteinuria and hypoalbuminemia. We conducted this observational study at our center to address these issues and contribute to the existing literature. The aim of this study is to analyze the clinical characteristics, histopathological findings, treatment responses, and long-term renal outcomes in patients with IgAN presenting with NS.

## Materials and methods

Patients

In this single-center, observational study, we retrospectively analyzed all the native kidney biopsies performed at our institute between January 2007 and December 2018. All cases of biopsy-proven IgAN, with a clinical presentation consistent with NS, were included. Exclusion criteria were age <14 years, diabetes, rapidly progressive renal failure, crescentic glomerulonephritis, diffuse global glomerulosclerosis, immunofluorescence showing trace or 1+ IgA staining, estimated glomerular filtration rate (eGFR) <30 ml/min per 1.73 m^2^, evidence of secondary causes of IgAN such as cirrhosis, any prior immunosuppressive therapy, and follow-up duration <6 months. The patients with nephrotic-range proteinuria but without hypoalbuminemia were also excluded.

Data collection

The baseline demographic, clinical, and pathological parameters were analyzed using electronic medical records. The data was retrospectively reviewed for age, sex, laboratory parameters, medications, follow-up duration, responsiveness to treatment, kidney functions, and proteinuria in the follow-up duration. Laboratory data included 24-hour urinary protein excretion, serum creatinine, albumin, and total cholesterol levels. The Chronic Kidney Disease Epidemiology Collaboration (CKD-EPI) equation was used to estimate GFR.

The patients were classified into three categories depending on their remission status at the last follow-up: complete remission (CR), partial remission (PR), and no response (NR).

Definitions

Nephrotic Syndrome (NS)

The presence of generalized edema, proteinuria of >3.5 g/day, hypoalbuminemia <3.5 g/dl, and/or hyperlipidemia.

Complete Remission (CR)

The absence of proteinuria (UPCR <0.3 g/g) along with the disappearance of edema, normalization of serum albumin, and stabilization of renal function.

Partial Remission (PR)

Greater than 50% reduction in proteinuria from baseline to <3.5 g/day.

No Response (NR)

Less than 50% reduction in proteinuria from baseline with or without deterioration of renal function.

Study outcomes

The study outcomes were absolute change in eGFR, progression to ESKD, or an eGFR-loss ≥15 ml/min through the follow-up period. ESKD was defined by either the requirement for dialysis or an eGFR of ≤15 ml/min.

Evaluation of kidney biopsies

The diagnosis of IgAN was made by the presence of dominant or co-dominant mesangial deposits of IgA on immunofluorescence microscopy. The biopsies with trace or 1+ IgA deposition were excluded. All biopsies were scored according to the Oxford MEST-C scoring and the Haas classification. Electron microscopy findings were unavailable for most patients and were not evaluated.

Treatment protocol

The patients included in the study received standard of care, including blood pressure management, maximally tolerated doses of an angiotensin-converting enzyme (ACE) inhibitor or angiotensin II receptor blocker (ARB), diet restriction, and lifestyle modifications. All patients were treated initially with oral steroids as per our center’s treatment protocol, with a starting dose of prednisolone 1 mg/kg/day (maximum 60 mg daily) tapered over a total of six months. The second-line agents, like mycophenolate mofetil and tacrolimus, were used in patients who did not achieve either complete or PR despite high-dose steroids for up to 16 weeks as tolerated. All patients on treatment with steroids (prednisolone ≥20 mg daily) received pneumocystis pneumonia prophylaxis using trimethoprim/sulfamethoxazole (TMP/SMX) 80/400 mg per day.

Ethical approval

The study was approved by the Institutional Ethics Committee of Sanjay Gandhi Postgraduate Institute of Medical Sciences, Lucknow, with Reference number 2020‑301‑DM‑EXP‑23, and conducted according to the principles of the Helsinki Declaration. Informed consent was waived due to the retrospective nature of the study.

Statistical analysis

All statistical analysis was performed using IBM SPSS software, version 25. Continuous variables were expressed as means, standard deviations, medians, and ranges and compared using an independent Student’s t-test or Mann-Whitney U‑test. Categorical and ordinal variables were expressed as frequencies and percentages and compared using the Chi-square or Fisher’s exact test. Statistical significance was defined as a p <0.05.

## Results

Five thousand, seven hundred fifty-one native kidney biopsies were performed at our center between January 2007 and December 2018; 681 (11.8%) biopsies were diagnostic of IgAN, and primary IgAN was present in 560 patients (9.7%). Out of 560 patients with primary IgAN, 67 (11.9%) patients with NS as the first clinical presentation were included in the study.

Baseline characteristics

The baseline characteristics of the included patients are shown in Table 1. Among these patients, 56 (83.6%) had generalized edema as the first presenting symptom, and only 1 (1.5%) patient had a history of gross hematuria. The mean age at the diagnosis was 27.67±10.37 years, and most patients were males (64.2%, n=43). The mean eGFR at initial presentation was 95.09±29.98 (range 39-154) ml/min/1.73 m^2^. The mean 24-hour proteinuria was 5.41±1.75 g/day. The mean serum albumin was 2.69±0.59 g/dl, and the median serum cholesterol was 268 mg/dl. Hypertension was seen in 25 (37.3%) cases with a mean systolic BP (SBP) of 131.37±16.55 mmHg and a mean diastolic BP (DBP) of 80.3±9.5. Nearly half of the patients (n=34, 50.7%) had microscopic hematuria at the baseline, but only 25.0% (n=3) of cases in Haas Class I had microscopic hematuria. Classes II, III, and IV had 54.5% (n=6), 50.0% (n=6), and 59.4% (n=19) cases of microscopic hematuria, respectively. AKI was present in 12 (17.9%) cases at the initial presentation.

**Table 1:** Baseline characteristics of all cases (n=67) ^#^Crescents >50% excluded AKI: Acute kidney injury; C: Crescents; DBP: Diastolic blood pressure; E: Endocapillary hypercellularity; eGFR: Estimated glomerular filtration rate; M: Mesangial hypercellularity; S: Segmental glomerulosclerosis; SBP: Systolic blood pressure; T: Tubular atrophy/interstitial fibrosis

Baseline characteristics	Value
Age, years, mean (± SD)	27.67 ± 10.37
Male, n (%)	43 (64.2%)
Hypertension, n (%)	25 (37.3%)
SBP, mmHg, mean (± SD)	131.37 ± 16.55
DBP, mmHg, mean (± SD)	80.30 ± 9.59
Gross hematuria, n (%)	1 (1.5%)
Laboratory measurements	
24-hour proteinuria, g/day, mean (± SD)	5.41 ± 1.75
Serum creatinine, mg/dl, mean (± SD)	1.05 ± 0.37
eGFR, ml/min, mean (± SD)	95.09 ± 29.98
Serum albumin, g/dl, mean (± SD)	2.65 ± 0.60
Serum cholesterol, mg/dl, median (IQR)	268 (208 – 340)
Microscopic hematuria, n (%)	34 (50.7%)
AKI, n (%)	12 (17.9%)
Follow-up, weeks, median (IQR)	190 (124 - 290)
Oxford classification (MEST-C score), n (%)	
M0	26 (38.8%)
M1	41 (61.2%)
E0	51 (76.1%)
E1	16 (23.9%)
S0	38(56.7%)
S1	29 (43.3%)
T0	58 (86.6%)
T1	9 (13.4%)
T2	0
C0	51 (76.1%)
C1	15 (22.4%)
C2^#^	1(1.5%)
Haas classification, n (%)	
I	12 (17.9%)
II	11 (16.4%)
III	12 (17.9%)
IV	32 (47.8%)
V	0

Renal biopsy findings

According to the Haas classification, most cases had class IV (diffuse proliferative) lesions (47.8%, n=32). On MEST-C scoring, mesangial hypercellularity (M1) was seen in 61.2%, endocapillary hypercellularity (E1) in 23.9%, segmental glomerulosclerosis (S1) in 43.3%, tubular atrophy/interstitial fibrosis (T1) in 13.4%, and crescents (C1) in 22.4% and (C2) in 1.5% cases (Table 1). No case had tubular atrophy/interstitial fibrosis involving >50% cortical area (T2) on biopsy. The baseline eGFR was significantly lower in the presence of segmental sclerosing lesions (S1) (p=0.04) and tubular atrophy/interstitial fibrosis involving >25% of cortical areas (T1/2) (p=0.004) (Table 2).

**Table 2:** Association between MEST-C score and baseline clinical variables ^#^Crescents >50% excluded *p-value <0.05 is statistically significant C: Crescents; E: Endocapillary hypercellularity; eGFR: estimated glomerular filtration rate; M: Mesangial hypercellularity; S: Segmental glomerulosclerosis; T: Tubular atrophy/interstitial fibrosis

	eGFR, mean (± SD)	*p-value*	24-hr proteinuria, mean (± SD)	*p-value*
Mesangial hypercellularity				
M0	94.50±35.39	0.90	4.90±1.28	0.06
M1	95.46±26.45		5.74±1.94	
Endocapillary hypercellularity				
E0	96.31±29.57	0.55	5.28±1.70	0.28
E1	91.19±31.89		5.83±1.93	
Segmental glomerulosclerosis				
S0	101.76±30.66	0.04*	5.32±1.95	0.63
S1	86.34±27.14		5.53±1.49	
Tubular atrophy/Interstitial fibrosis				
T0	99.16±28.29	0.004*	5.25±1.65	0.05
T1/2	68.89±28.58		6.47±2.13	
Crescents				
C0	99.04±28.39	0.05	5.18±1.56	0.06
C1/2^#^	82.50±32.32		6.14±2.17	

Clinical features of patients according to clinical response

All included patients except one were treated with steroids, and 33 patients achieved remission, either complete (n=14) or partial (n=19) with steroids only. Second-line agents, mycophenolate mofetil, tacrolimus, or its combination, were used in 29 patients, and among these, 14 achieved complete and 12 achieved PR. Despite immunosuppression, seven (10.4%) patients did not achieve remission. Table 3 shows the clinical features of patients according to clinical response. No significant difference was observed in age (p=0.96), gender (p=0.13), and blood pressure (p=0.94) between the three groups. At baseline, the mean eGFR (p=0.36), 24-hour proteinuria (p=0.66), and albumin (p=0.35) were similar across the groups.

**Table 3:** Clinical features and outcomes of patients according to clinical response ^#^Crescents >50% excluded *p-value <0.05 is statistically significant C: Crescents; CNI: Calcineurin Inhibitors; E: Endocapillary hypercellularity; eGFR: Estimated glomerular filtration rate; ESKD: End-stage kidney disease; M: Mesangial hypercellularity; MMF: Mycophenolate Mofetil; S: Segmental glomerulosclerosis; T: Tubular atrophy/interstitial fibrosis

	Complete remission (n = 28)	Partial remission (n = 32)	No response (n = 07)	*p-value*
Age, years, mean (± SD)	28.11 ± 9.05	27.38 ± 11.45	27.29 ± 11.63	0.96
Male, n (%)	14 (50%)	23 (71.9%)	6 (85.7%)	0.13
Hypertension, n (%)	11 (39.3%)	12 (37.5%)	2 (28.6%)	0.94
Oxford classification (MEST-C score), n (%)				
M0	13 (46.4%)	10 (31.25%)	3 (42.9%)	0.51
M1	15 (53.6%)	22 (68.75%)	4 (57.1%)	
E0	20 (71.4%)	25 (78.1%)	6 (85.7%)	0.71
E1	8 (28.6%)	7 (21.9%)	1 (14.3%)	
S0	17 (60.7%)	17 (53.1%)	4 (57.1%)	0.88
S1	11 (39.3%)	15 (46.9%)	3 (42.9%)	
T0	24 (85.7%)	29 (90.6%)	5(71.4%)	0.33
T1/2	4 (14.3%)	3 (9.4%)	2 (28.6%)	
C0	21 (75.0%)	24 (75.0%)	6 (85.7%)	1.00
C1/2^#^	7 (25.0%)	7 (31.2%)	1 (14.3%)	
Haas classification, n (%)				
I	7 (25.0%)	5 (15.6%)	0	0.29
II	3 (10.7%)	6 (18.7%)	2 (28.6%)	
III	7 (25.0%)	3 (9.4%)	2 (28.6%)	
IV	11 (39.3%)	18 (56.2%)	3 (42.9%)	
V	0	0	0	
Follow-up, weeks, median (IQR)	221 (137 - 310)	159 (112 - 283)	190 (140 - 364)	0.33
Laboratory parameters at baseline				
24-hour proteinuria, g/day, mean (± SD)	5.23 ± 1.54	5.45 ± 1.72	5.90 ± 2.70	0.66
Serum Creatinine, mg/dl, mean (± SD)	1.08 ± 0.40	0.99 ± 0.28	1.24 ± 0.55	0.24
eGFR, ml/min, mean (± SD)	90.79 ± 29.57	100.53 ± 27.87	87.43 ± 40.28	0.36
Serum Albumin, g/dl, mean (± SD)	2.61 ± 0.64	2.73 ± 0.54	2.39 ± 0.67	0.35
Microscopic hematuria, n (%)	14 (50%)	17 (53.1%)	3 (42.9%)	0.94
Laboratory parameters at the last follow-up				
Serum Creatinine, mg/dl, mean (± SD)	1.06 ± 0.35	1.47 ± 1.82	4.46 ± 4.71	<0.001*
eGFR, ml/min, mean (± SD)	89.25 ± 27.78	85.87 ± 33.55	52.14 ± 44.67	0.03*
Type of immunosuppressants, n (%)				
Steroids only	14 (50%)	19 (59.4%)	4 (57.1%)	0.66
Steroids + MMF	11 (39.3%)	9 (28.1%)	1 (14.3%)	
Steroids + CNI	2 (7.1%)	2 (6.2%)	1 (14.3%)	
Steroids + MMF + CNI	1 (3.6%)	1 (3.1%)	1 (14.3%)	
Long-term clinical outcomes				
∆eGFR, median (IQR)	2.5 (-16.75 to 24.00)^α^	11.5 (-3.5 to 34.50)^β^	34.0 (22.0 to 45.0)^ϒ^	α vs β = 0.14, α vs ϒ = 0.002*, β vs ϒ = 0.02*
eGFR-loss ≥15 ml/min, n (%)	9 (32.1%)	15 (46.9%)	7 (100.0%)	0.004*
ESKD, n (%)	0	1 (3.1%)	3 (42.9%)	0.003*

Only two patients (3.0%) in the CR group relapsed during follow-up, but both attained remissions again with immunosuppression. The mean eGFR at the last follow-up in CR, PR, and NR groups was 89.25±27.78, 85.87±33.55, and 50.43±41.61, respectively (p=0.03).

Long-term clinical outcomes

During the median follow-up of 190 weeks (~3.6 years), the median absolute change in eGFR (∆eGFR) was significant between CR and NR groups (p=0.002), and PR and NR groups (p=0.02); however, it was not significant between CR and PR groups (p=0.14). In the CR, PR, and NR groups, 9, 15, and 7 patients had eGFR losses of ≥15 ml/min (p=0.004). One in the PR group and three in the NR group progressed to ESKD, while no patient in the CR group progressed to ESKD (p=0.003) (Table 3).

Association between MEST-C scores and clinical outcome

Table 4 shows the association between MEST-C scores and clinical outcomes of the patients. However, none of the scores correlated with the clinical outcome parameters.

**Table 4:** Association between MEST-C score and clinical outcomes ^#^Crescents >50% excluded *p-value <0.05 is statistically significant C: Crescents; E: Endocapillary hypercellularity; eGFR: Estimated glomerular filtration rate; M: Mesangial hypercellularity; S: Segmental glomerulosclerosis; T: Tubular atrophy/interstitial fibrosis

	M0 (n=26)	M1 (n=41)	*p-value*	E0 (n=51)	E1 (n=16)	*p-value*	S0 (n=38)	S1 (n=29)	*p-value*	T0 (n=58)	T1/2 (n=9)	*p-value*	C0 (n=51)	C1/2^#^ (n=16)	*p-value*
Complete remission	13 (50.0%)	15 (36.6%)	0.32	20 (39.2%)	8 (50.0%)	0.56	17 (44.7%)	11 (37.9%)	0.62	24 (41.4%)	4 (44.4%)	1.00	21 (41.2%)	7 (43.7%)	1.00
eGFR-loss ≥15 ml/min	12 (46.1%)	19 (46.3%)	1.00	24 (47.1%)	7 (43.7%)	1.00	15 (39.5%)	16 (55.2%)	0.23	27 (46.6%)	4 (44.4%)	1.00	26 (51.0%)	5 (31.2%)	0.25
ESKD	2 (7.7%)	2 (4.9%)	0.64	4 (7.8%)	0	0.56	3 (7.9%)	1 (3.4%)	0.63	2 (3.4%)	2 (22.2%)	0.08	3 (5.9%)	1 (6.2%)	1.00

## Discussion

In this study, we observed that 11.9% of patients with primary IgAN had initial presentation consistent with NS. We also found that appropriate immunosuppressive treatment led to remission of proteinuria, either complete or partial, in the majority of patients. The patients who achieved remission even partially had favorable long-term renal outcomes. Further on MEST-C scoring, the baseline eGFR was significantly lower in the presence of S1 and T1/2 lesions; however, none of the scores correlated with the outcome parameters.

IgAN most commonly presents with microscopic hematuria and varying degrees of proteinuria, with or without renal impairment [5]. In a previous study of IgAN patients from our center, Prasad et al. reported a median eGFR of 34.29 ml/min/1.73 m^2^ (range 3.25-160.4) and a median serum creatinine level of 2.3 mg/dl (range 0.5-17.5) [16]. We observed that our subset of patients with NS had a higher eGFR and a lower serum creatinine at presentation. As expected, these patients had lower serum albumin and higher serum cholesterol levels. Fewer patients in our study presented with hypertension compared to the findings of Prasad et al. (37.3% vs 69.8%) [16]. The higher eGFR and fewer cases of hypertension in our cohort may be attributed to the early onset of symptomatic edema, which makes these patients seek medical attention at the initial stage of the disease and have a kidney biopsy done. Microscopic hematuria was observed in nearly half of our patients (n=34, 50.7%), compared to 63.2% in the previous study [16]. Only 25.0% of cases in Haas Class I had microscopic hematuria, which is consistent with the minimal histologic changes observed in this class.

The previous studies reporting NS in IgAN had small numbers of cases with minimal histologic lesions and normal renal function, with an excellent response to steroids [7,13,14,17]. However, nephrotic-range proteinuria may occur in any subclass of IgAN, and patients with advanced pathologic stages may not respond well to steroids. Thus, NS presentation in IgAN is not entirely similar to MCD as previously thought. In our study, proliferative (Class IV (47.8%, n = 32), Class III (17.9%, n = 12)) as well as segmental sclerosing (Class II (16.4%, n = 11)) lesions were seen as determined by the Haas classification. All these patients received some form of immunosuppression, and most achieved remission, completely or partially. Even patients with relatively severe proliferative lesions on biopsy could achieve remission with immunosuppression.

Another significant finding in this study was that the prognosis was better in patients who achieved remission. Even the patients who achieved PR had better eGFR and less progression to ESKD than those who did not. Reich et al. reported that patients with IgAN who initially presented with proteinuria >3 g/d and achieved PR (<1 g/d) had similar outcomes to patients with persistent proteinuria of <1 g/d during follow-up and also had superior prognoses to patients who never achieved remission [18]. However, that study included all patients with IgAN regardless of kidney function and proteinuria status. Kim et al. demonstrated that in a cohort of 100 biopsy-proven IgAN patients with NS and a baseline eGFR of 69.4 ± 17.5 ml/min, the prognosis was unfavorable unless partial or CR was achieved [8]. Similarly, our findings support the significance of achieving remission of proteinuria, whether partial or complete, in improving renal outcomes for this specific subgroup of patients with IgAN and NS.

The Oxford classification of IgAN defines histopathologic scores (MEST-C) that provide prognostic information based on the kidney biopsy [19]. Recent studies have demonstrated that the MEST-C scores could be utilized to predict prognosis in IgAN [20-24]. Schimpf et al., in the secondary analysis of STOP-IgAN biopsies, observed that mesangial hypercellularity (M1) was associated with higher annual eGFR-loss, and tubulointerstitial fibrosis (T1/2) was associated with lower baseline eGFR and ESKD development [25]. We observed that baseline eGFR was significantly lower in patients with S1 and T1/2 scores, as expected since segmental sclerosing lesions and tubular atrophy/interstitial fibrosis represent advanced irreversible kidney damage regardless of the underlying pathology. However, we found no correlation between MEST-C scores and the analyzed outcomes parameters. This could be because of the responsiveness of M1 and E1 lesions to immunosuppressive therapy, the exclusion of crescentic glomerulonephritis and diffuse global glomerulosclerosis, as well as the lack of severe interstitial fibrosis and tubular atrophy in the study cohort. Further, the limited follow-up duration of 3.6 years and the overall low number of ESRD events may also have influenced the outcomes.

Our study has certain limitations. The study’s retrospective design and single-center setting limit the generalizability of our findings. There is no consensus on whether NS in IgAN is an MCD with incidental IgA deposition or a true IgAN. However, previous studies have suggested that the former is more likely when biopsy findings show a class I lesion by the Haas classification, diffuse foot process effacement, and trace or 1+ IgA deposition [26,27]. Although we did not include the patients with trace or 1+ IgA deposition, the electron microscopy findings were also not assessed. There was no fixed protocol for using second-line agents, like mycophenolate mofetil and tacrolimus, which was at the treating physician’s discretion. We did not evaluate the impact of the renin-angiotensin system (RAS) and Sodium-Glucose Transport Protein 2 (SGLT2) inhibitors, which have become increasingly relevant in IgAN treatment [28-30]. Although it is unlikely that either agent alone can achieve CR, some patients may be able to achieve PR with immunosuppressants in combination with these medications. We used ACE inhibitors or ARBs in the maximum tolerated dose per treatment protocol. However, the effectiveness of SGLT-2 inhibitors in reducing proteinuria was not established during our study period.

## Conclusions

We observed that immunosuppression can be beneficial in treating NS associated with IgAN and achieving remission, whether complete or partial, is essential for improving renal survival. At the very least, PR should be attained to delay the disease progression. Furthermore, according to the MEST-C classification, the baseline eGFR was significantly lower in cases with S1 and T1/2 lesions in kidney biopsies. Thus, our findings may have therapeutic implications for the management of patients with IgAN presenting with NS. However, further prospective multicenter studies are needed to confirm these results and establish definitive treatment recommendations.
